# S-MART, A Software Toolbox to Aid RNA-seq Data Analysis

**DOI:** 10.1371/journal.pone.0025988

**Published:** 2011-10-06

**Authors:** Matthias Zytnicki, Hadi Quesneville

**Affiliations:** URGI, INRA, Versailles, France; University of Minnesota, United States of America

## Abstract

High-throughput sequencing is now routinely performed in many experiments. But the analysis of the millions of sequences generated, is often beyond the expertise of the wet labs who have no personnel specializing in bioinformatics. Whereas several tools are now available to map high-throughput sequencing data on a genome, few of these can extract biological knowledge from the mapped reads. We have developed a toolbox called S-MART, which handles mapped RNA-Seq data. S-MART is an intuitive and lightweight tool which performs many of the tasks usually required for the analysis of mapped RNA-Seq reads. S-MART does not require any computer science background and thus can be used by all of the biologist community through a graphical interface. S-MART can run on any personal computer, yielding results within an hour even for Gb of data for most queries. S-MART may perform the entire analysis of the mapped reads, without any need for other *ad hoc* scripts. With this tool, biologists can easily perform most of the analyses on their computer for their RNA-Seq data, from the mapped data to the discovery of important loci.

## Introduction

High-throughput sequencing through next-generation sequencing technologies has dramatically expanded the number of experiments made by sequencing. Today, almost all life-science fields are affected by these developments. The latest sequencers now provide about 100Gb of data per run making computer-aided analysis compulsory. Several software packages have been developed to map the reads onto a reference genome (*e.g.* MAQ [1], BWA [2], [3], SOAP2 [4], BowTie [5] or Mosaik [6]). However after the mapping, the user gets a huge set of genomic coordinates, which remain to be analyzed.

Several pipe-lines have already been developed for the analysis of RNA-Seq data for the discovery of genes [7], miRNAs [8], or piRNAs [9]. However, an experiment does not usually follow a rigid set of bioinformatic tasks and the user usually adapts the analysis according to preliminary results. In this case, the biologist usually requires the help of a bioinformaticians to conduct the analysis.

S-MART is a versatile toolbox which can perform most RNA-Seq analysis, although it is not a pipe-line *per se*. S-MART performs two categories of tasks: data manipulation and visualization. Manipulations include (i) selection/exclusion of the reads which overlap with some reference sets, (ii) read clustering, and (iii) differential expression analysis. Visualizations include (i) read-size distribution, (ii) nucleotidic compositions, (iii) chromosome localizations, and (iii) distances with respect to a reference set.

A particular effort has been made for biologists with little background in computer science. A graphical user interface allows the user to launch every tool simply by pressing buttons. S-MART is available on Windows, Mac, and Linux platforms.

The major advantage of S-MART over other tools (*e.g.* Galaxy [10]) is that the whole RNA-Seq analysis can be performed on any computer (even a laptop with limited resources), and on any OS (because some mapping tools like BowTie are available on any OS).Furthermore, S-MART is intuitive and easy to use, even for people with no computer-science background.

Finally, S-MART provides a wide list of useful tools which are commonly used for RNA-Seq analysis. Although some of the tools that S-MART provides are available in other software packages, S-MART offers a unified, simple, and synthetic framework for the analysis of RNA-Seq data. We expect that many questions involving RNA-seq data can be answered with current version of S-MART. Software will be under continually enhancement.

## Results

S-MART performs different categories of tasks. First, it can (i) filter and select the data of most interest, (ii) cluster the information to acquire a bird's eye view, or (iii) convert the data from one file format to another. Second, it can (i) produce high-quality graphs to visualize some aspects of the information from the reads, or (ii) plot some general distributions. Third, S-MART can discriminate the differentially expressed genes (or any annotation).

S-MART has been used on Illumina and Roche data. It seamlessly handles large sets of data (such as Illumina) and long reads (such as Roche) which may contain introns. It has been successfully applied to our own Illumina Genome Analyzer and Roche Genome Sequencer.

### Operations

#### Filtering

S-MART can read output files from many mapping tools. It can then select the mappings following different criteria: with/without mismatches, with only one or several matches on the genome, *etc.*

S-MART can also compare read genomic coordinates with a reference set of annotations. Annotations can be coding-gene annotations (*e.g.* RefSeq), transposable elements, miRNAs, *etc.* The user can therefore easily compute the number of reads which were produced by his annotation of interest. S-MART may also compute overlaps with flanking regions, *e.g.* to obtain the reads produced by promoter regions.

#### Clustering

S-MART can merge overlapping mapped reads into clusters or gather them using a user defined window. Overlapping data can also be merged to find more “exotic” patterns such as double-strand transcriptions or putative bidirectional promoters.

#### Conversion

S-MART includes several other tools which may help the user: file format converter, genomic coordinates modifier, *etc.*

### Visualization

#### Read information

Different plots can be produced in PNG files showing (i) the number of times a sequence has been obtained, (ii) the size of the reads, (iii) the number of exons per read or reads per cluster, and (iv) the nucleotidic composition.

#### Distributions

Several distributions can be extracted from the mapped reads, or any set of genomic coordinates. These include (i) the density on the chromosomes, (ii) the read distance with respect to a reference set (*e.g.* RefSeq data), or (iii) other general correlations.

S-MART produces standard GFF3 files by default, but it can also export the data in a format which can be loaded into UCSC genome browser using the BED format [11], or by any Gbrowse [12], using their specific annotation file format. It is thus possible for the user to visualize his/her data through any genome browser.

#### Comparison with epigenomic ChIP-Seq data

S-MART can also plot epigenomic ChIP-Seq or MNase-Seq data (such as nucleosome positioning, histone modification, methylation, *etc.*), in comparison with annotation or RNA-Seq data. For instance, S-MART can plot the average level of some histone modification along a given gene, or plot the average level of histone modification around the transcription start site of the gene.

### Differential expression

Many papers use RNA-Seq data to identify differentially expressed genes (or regions), typically between wild-type and mutant conditions. Different statistical techniques have been developed to identify differentially expressed genes. S-MART contains an out-of-the box way to compare the two conditions using a Fisher's exact test. Any set of genomic regions, including transposable elements, miRNAs, *etc.*, can be used as the reference set instead of a set of genes.

Several normalizations and adjustments are available. The first one is the simplest: the number of reads in each condition is normalized to the average number of reads. Bullard *et al.* showed that this normalization is problematic when the most expressed regions are differentially expressed [13]. The second normalization supposes that moderately expressed genes are not differentially expressed. It sorts the genes according the average number of reads under the two conditions and normalizes the two samples so that the genes in the interquartile range (the difference between the first and third quartiles) have the same number of reads.

Oshlack and Wakefield have also shown that this method is biased because the longest genes tend to be more significantly differentially expressed, simply as a result of matching more reads, thereby increasing the power of the test [14]. The third possible normalization finds the size of the smallest gene, and then uses this as the size of a sliding window. Then, instead of counting the reads in each gene, S-MART uses the sliding window from the 5′ to the 3′ part of the gene and counts the average number of reads.

Finally, a false discovery rate (FDR) filter can be used to select differentially expressed regions of candidates.

### Example of a pipe-line

**Figure 1:**
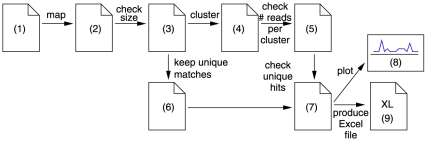
A pipe-line for the detection of piRNA clusters, using S-MART.

To illustrate the usefulness of S-MART, we present how its different tools can be combined to perform an analysis in Figure 1.

The Piwi-interacting RNAs (piRNAs) are RNAs of 28- to 30-nucleotides in length, which form RNA-protein complexes with Piwi proteins. These piRNA complexes have been linked to transcriptional silencing of transposable elements in germ-line cells from animals. Brennecke J *et al.*have presented a pipe-line to discover the clusters of piRNAs using ChIP-Seq data [9].

Suppose that we have a set of reads obtained from immuno-precipitation of mouse Piwi (file 1) and we want to find the clusters of piRNAs. We have mapped them to the genome, by using BowTie (file 2).

We first remove the reads of sizes greater than 30, or less than 28, to exclude other small RNA (file 3). We cluster the data and since piRNAs usually gather in clusters (file 4), and we keep the clusters with at least 10 reads (file 5).

Since some piRNAs target transposable elements, it is possible that a cluster contains only non-unique mapping reads. This is problematic because we are not sure that the cluster is actually present at this locus. To solve this problem, we can extract all of the reads that mapped only once (file 6) and keep the clusters which contain at least one such read (file 7). The clusters can finally be plotted along the chromosome (file 8), and the list can be written in an Excel file (file 9).

## Discussion

### Design and Implementation

Most current RNA-Seq data give over 20 million reads from one experiment. When the reads have been mapped, handling such a large set of genomic coordinates is a non-trivial algorithmic problem. The algorithms should be fast enough to run within a reasonable amount of time. Then, since S-MART should be run on a standard computer with a limited RAM, extra care has to be brought to use the minimum amount of memory. To solve these problems, we store the data into SQLite tables, and use nested bins to retrieve the overlapping genomic coordinates efficiently as performed by Kent *et al*. [11]. A B-tree index is used on the nested bins to speed up the search.

The algorithms have been implemented as a library of Python objects modeling the mapped sequences and the genomic annotations to be compared — these can be transcripts, transposable elements, transcription factor binding sites *etc*. The implementation handles the sets of genomic coordinates as SQLite entries to efficiently perform both low level (as the simple modification of genomic coordinates) and high level (as the distance between two transcripts) operations on the sets of data. The toolbox includes an overlap detection engine, a driver for graphical outputs, as well as parsers for many formats such as: (i) AXT, Blast *-m 8*, Blat, MAQ, Mummer, Nucmer, PSL, Rmap, SAM, SeqMap, Shrimp, SOAP for the mappers formats; (ii) BED and GFF for the annotation formats. S-MART can also convert data to different output formats, including GFF3, BED, and SAM formats, an Excel-compatible format, and other formats that can be loaded into the most commonly used genome browsers: GBrowse or UCSC Genome Browser.

The graphical user interface has been written in Java. S-MART can be used on Windows, Mac and Linux operating systems, as long as the tools needed by S-MART —which are freely available— are installed (namely, Python, Java, R).

### Availability and Future Directions

S-MART can be downloaded for free from [15], and can be used on any platform (Linux, Windows or Mac). S-MART has been developed under the CeCILL license, which is compatible with the GNU GPL license.

In the future, we will take advantage of the modularity of the toolbox to add new functionalities. We are also currently porting S-MART modules to Galaxy, in particular those that bring functionalities missing from Galaxy.
